# Atherosclerosis: Molecular Pathogenesis and Therapeutic Interventions

**DOI:** 10.1002/mco2.70857

**Published:** 2026-07-13

**Authors:** Jonatan Kaszubski, Agata Wawrzyniak, Maciej Gagat, Agnieszka Żuryń

**Affiliations:** ^1^ Vascular Biology Student Research Club Department of Histology and Embryology Faculty of Medicine Collegium Medicum in Bydgoszcz Nicolaus Copernicus University in Toruń Bydgoszcz Poland; ^2^ Department of Histology and Embryology Institute of Medical Sciences College of Medical Sciences of the University of Rzeszów University of Rzeszów Rzeszów Poland; ^3^ Department of Histology and Embryology Faculty of Medicine Collegium Medicum in Bydgoszcz Nicolaus Copernicus University in Toruń Bydgoszcz Poland

**Keywords:** atherosclerosis, CDK5, CDK9, miRNA, PROTACs, siRNA

## Abstract

Atherosclerosis is a multifactor condition and driving force behind many cardiovascular diseases. Moreover, it remains a leading cause of mortality worldwide. The current therapies, including statins and fibrates, focus mainly on lipid lowering. However, this condition is characterized by various biological pathways, particularly the oxidized LDL uptake by macrophages, chronic inflammation, or vascular smooth muscle cells proliferation, migration, and phenotypic switching, which forces the researchers to explore the numerous molecular pathways standing behind those processes. Despite a lot research on this topic was conducted, the need of finding new therapeutic methods based on the molecular pathogenesis still remains. In this review, we summarize the involvement of the lead signaling pathways in atherosclerosis: transforming growth factor beta (TGF‐β), mitogen‐activated protein kinase (MAPK) and phosphoinositide 3‐kinase/protein kinase B (PI3K/Akt), as well as the functions of cell cycle proteins in its pathogenesis. We are going to put an emphasis on the cyclin‐dependent kinases (CDKs) 5 and 9, as the driving forces behind the pathological conditions. Furthermore, we describe the novel therapeutic interventions, including noncoding RNA strategies and proteolysis targeting chimeras (PROTACs), as they are important candidates for future therapies. Our insights may help in the thorough understanding of the atherosclerosis pathogenesis and finding the future therapeutic interventions.

## Graphical abstract

Molecular factors, like transforming growth factor beta (TGF‐β), mitogen‐activated protein kinase (MAPK), phosphoinositide 3‐kinase (PI3K), or cyclin‐dependent kinases (CDKs) 5 and 9, are essential modulators of atherosclerotic lesions. The novel, promising strategies for atherosclerosis treatment include therapies based on small interfering RNA (siRNA), microRNA (miRNA) targeting, long noncoding RNA (lncRNA), and proteolysis targeting chimeras (PROTACs).

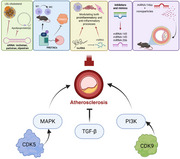

## 1 Introduction

Cardiovascular diseases (CVDs) affect more than half of billion people worldwide and constitute a leading cause of mortality [1]. Coronary artery disease (CAD) is considered the most frequent form of CVD and its most important driving factor is atherosclerosis—a multifactor pathology process affecting various cell among the arterial wall and involving many biological mechanisms that have been discovered over the years. The most characteristic changes within the vessel wall in atherosclerosis are mostly described as chronic inflammation and lipid metabolism disfunction [2, 3, 4]. An important sign of atherosclerosis is a dysregulated proliferation, migration, and phenotypic switching of vascular smooth muscle cells (VSMCs) [2]. Phenotypic switching from contractile to synthetic VSMCs often leads to increased proteolytic enzymes production and eventually to VSMCs apoptosis [5]. There is a growing interest of the involvement of transforming growth factor‐β (TGF‐β), a common inflammation regulator factor, as well as various microRNAs (miRNAs) and Krüppel‐like factor 4 [6]. Noncoding, counting only 18–25 nucleotides miRNAs are significantly interesting, especially miRNAs‐143/145 or miRNA‐193‐3p, as they appear to be specific biomarkers of VSMCs phenotypic switching [6, 7]. There were also reports that switching to the synthetic phenotype is mediated by the small GTPase RhoA and its effector kinase, Rho‐associated kinase [8]. What is worth noting is that this process is also escalated by reactive oxygen species (ROS), which in very high level may lead to the oxidative stress and dysregulation in the proper VSMCs functioning [9]. Those pathological processes are deeply controlled by a network of signal pathways, including TGF‐β, mitogen‐activated protein kinase (MAPK), and phosphoinositide 3‐kinase (PI3K)/protein kinase B (Akt)/mammalian target of rapamycin (mTOR) [10].

About 30 years ago, the topic of the cell cycle role in VSMCs proliferation has started to be discovered [11]. The cell cycle is controlled by the group of serine/threonine proteins called cyclin‐dependent kinases (CDKs) and other their binding proteins called cyclins. Their natural inhibitors, especially p27^KIP1^, p21^CIP1^, and p16^INK4a^, have become a key target of research on the dysfunctional proliferation of VSMCs [11, 12]. Although the role of classic CDKs, which control the cell cycle directly, has been described extensively, the number of studies dedicated to “untypical” CDKs is very scant. However, some of those kinases may play a key role in various mechanisms in the pathogenesis of atherosclerosis like lipoprotein uptake by macrophages, endothelium senescence, or phenotypic switching of VSMCs [13, 14, 15, 16]. They involve CDK5, an unusual CDK that is mostly known for its regulating role in nervous system, as well as CDK7/8/19/9, which are classified as CDKs responsible for controlling the transcription process [17].

**FIGURE 1:**
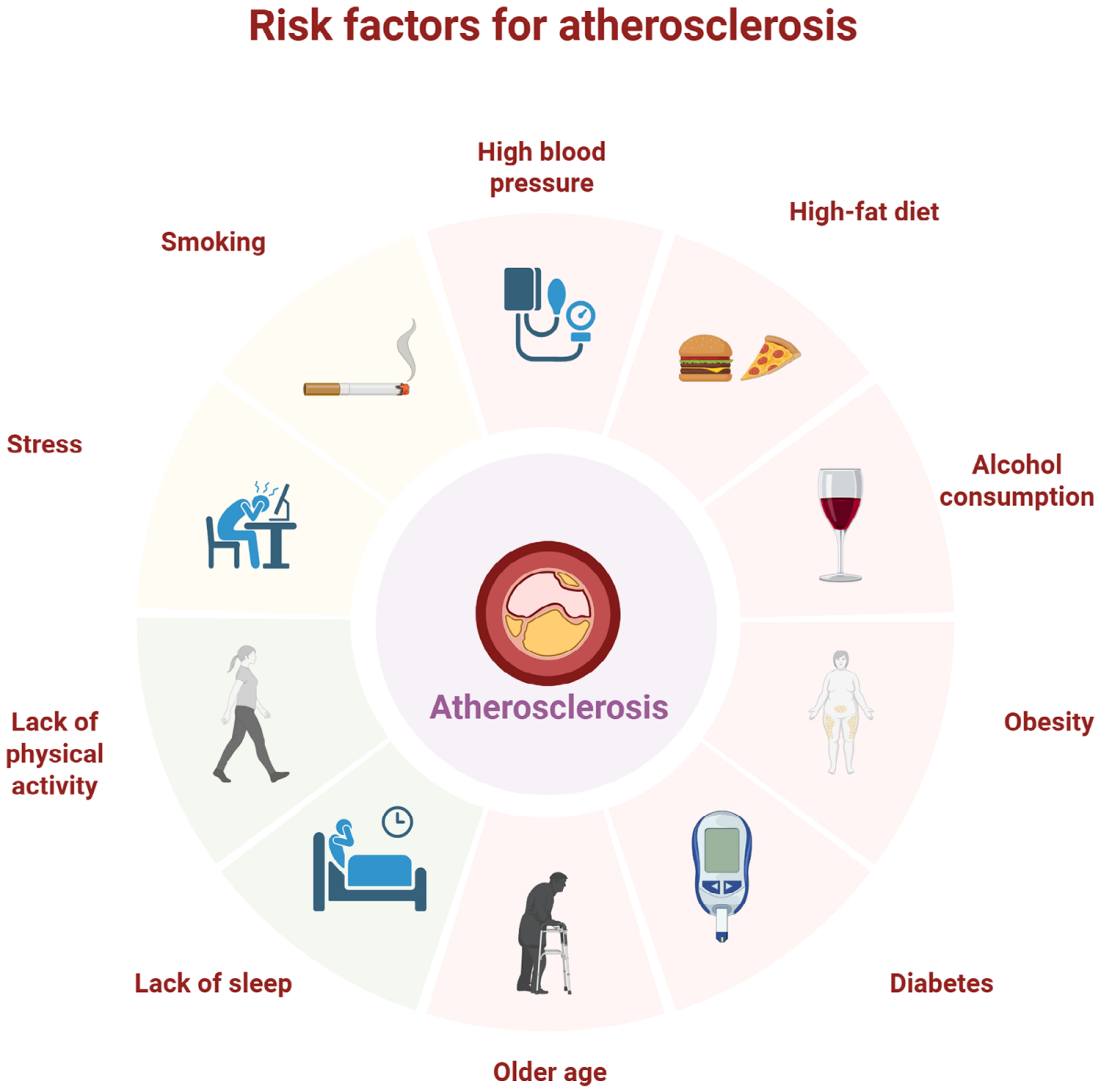
The atherosclerosis development is often connected to the occurrence of various environmental risk factors. The main factors, which affect the changes within the arteries, include high blood pressure, high‐fat diet, stress, obesity, diabetes, and older age. The research show that smoking, alcohol consumption, as well as lack of sleep or physical activity, highly increase the risk of the atherosclerosis development. The unhealthy lifestyle frequently causes the abnormal blood cholesterol levels and therefore increases the risk of plaque formation (illustration created by BioRender.com).

It is very important that not only genetic factors stand behind the atherosclerosis progression. The environment has also been proved to strongly affect the condition of cardiovascular system. According to research, the unhealthy conditions, like high blood pressure, high‐fat or high‐sugar diet, obesity, as well as the lack of sleep and physical activity, are the common risk factors causing the CVDs. Moreover, people with Type‐2 diabetes or older age also have been found to have a higher risk of atherosclerosis development [3, 18]. The most common risk factors were presented in the Figure 1.

Classic treatment options for atherosclerosis include statins and fibrates; however, they mainly focus on lipid lowering. The complexity of atherogenesis forces us to look for new therapeutic methods, targeting factors involved in the inflammatory response and VSMCs proliferation. The novel interesting therapeutic interventions, which can be used in therapy in the future, include noncoding RNA interventions and protein‐degrading proteolysis targeting chimeras (PROTACs) [19].

In the purpose of writing this article, we searched the PubMed database, in order to find the recent studies on the novel therapeutic interventions in the atherosclerosis treatment. In this review, we are going to summarize the basic information of atherosclerotic lesions and well‐known signal pathways involved in their progression. Next, we are going to describe a potential role of rare and transcriptional CDKs in the progression of atherosclerosis in detail, as it was not comprehensively described before. We will also emphasize the new therapeutic methods, like PROTACs or small interfering RNA (siRNA) delivery, as they seem to be very promising strategies for the novel treatment.

## 2 Molecular Pathogenesis of Atherosclerosis

**FIGURE 2:**
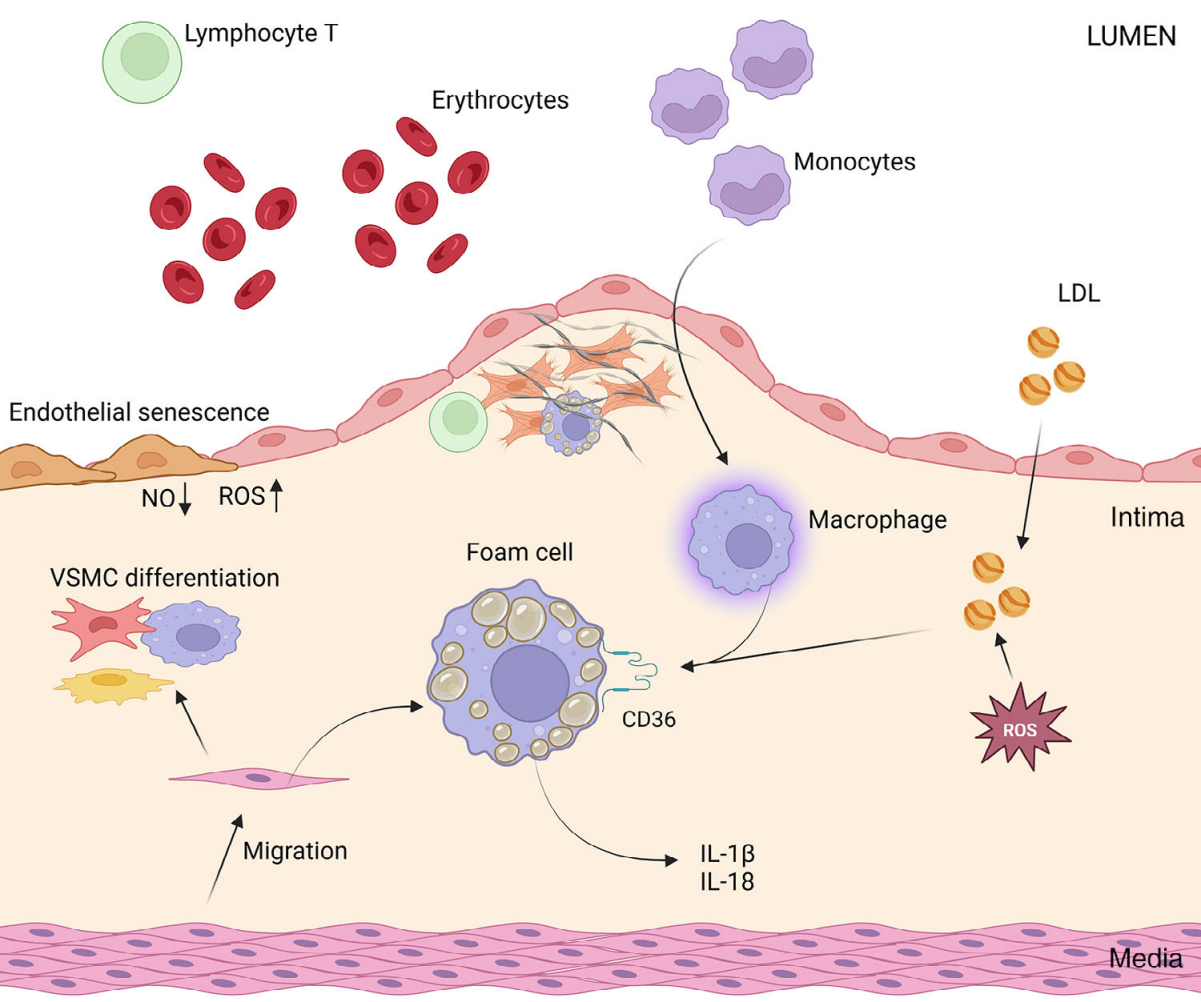
The pathogenesis of atherosclerosis include various molecular pathways and is represented by many cells existing within the artery wall. The low‐density lipoprotein (LDL) migrate through the dysfunctional epithelium. In response, the monocytes migrate through the epithelium and transform themselves into the macrophages. The senescence of the epithelium causes the reduction of nitro oxygen production and increases the production of reactive oxygen species (ROS), which enables the process of LDL oxidation. The uptake of the oxidized LDL (ox‐LDL) by the macrophages transforms them into the foam cells, which play a crucial role in the plaque formation. The migration of vascular smooth muscle cells (VSMCs) and its phenotypic switching into the macrophage‐like VSMCs, osteoblast/chondrocyte‐like VSMCs, or mesenchymal‐like VSMCs increases the process of foam cell formation. The foam cells starts producing the proinflammatory cytokines, including IL‐1β and IL‐18, which eventually leads to the chronic inflammation within the artery wall (illustration created by BioRender.com).

Atherosclerotic lesions are concerned around the changes within the large arteries, which mainly consist of a chronic inflammation process, a disfunction of endothelium, lipid accumulation, and oxidation within the arterial wall [2, 3]. The basic molecular pathogenesis of atherosclerosis is presented in Figure 2.

One of the first stages of atherosclerosis is the endothelial disfunction, frequently observed earlier than other symptoms. Whereas properly‐functioning endothelial cells form a single tight layer and regulate the tone and structure of blood vessels by releasing various substances like nitric oxide (NO), a dysfunctional endothelium; a frequent consequence of NO activity or production defect causes structural damage of the vessel wall and promotes the uptake of low‐density lipoprotein (LDL) [3, 20]. LDL consists mainly of cholesterol, apolipoprotein B‐100 (ApoB‐100) and apolipoprotein E (ApoE). They are created from the very LDL that is responsible for transporting cholesterol and fatty acids from hepatocytes to other tissues. After transforming into LDL, lipoproteins are able to penetrate through the damaged endothelium to the tunica intima, where they are very susceptible to the oxidation process by ROS [3, 4, 10]. In the blood vessels, ROS are produced mainly by those enzymatic pathways where the electrons are transported to the oxygen molecules, including mitochondrial electron transport chain, NADPH oxidases, xanthine oxidase, or endothelial nitric oxide synthase. The optimal level of ROS is needed to maintain the proper functioning of the vascular system. However, when the antioxidative systems are damaged or the ROS level is too high, we deal with a state called oxidative stress. ROS are able to react with various molecules, including LDL, and therefore exacerbates atherosclerotic lesions [21]. NOX1, the important isoform of NADPH oxidase, was lately described to play an important role in CVD. Its deletion in apolipoprotein E‐deficient (ApoE−/−) mice and in ApoE−/− mice after induction of diabetes was found to significantly reduce the oxidative stress, macrophage infiltration, and the lesion area [21, 22, 23].

The monocytes react on the process of LDL infiltration in the tunica intima and transform themselves into M1 macrophages. After oxidized LDLs (ox‐LDLs) are taken up by macrophages, the cholesterol engorged macrophages, also known as “foam cells,” are created. The uptake of ox‐LDL is driven by scavenger receptors like CD36, which are able to bind ox‐LDL and mediately activate the nuclear factor‐kappa B (NF‐κB). NF‐κB is next translocated to the nucleus, where it upregulates the transcription of proinflammatory cytokines like IL‐1β and IL‐18, which lead to releasing the dendritic cells, as well as lymphocytes T and B; therefore, the inflammation is escalated [4, 10].

The foam cells, lymphocytes T, and VSMCs are slowly starting to accumulate into the atherosclerotic plaque [4]. VSMCs are located in the tunica media, but they can migrate to tunica intima and become a dominant component of the plaque. In atherosclerosis, VSMCs are characterized by increased proliferation and phenotypic switching to various phenotypes including macrophage‐like VSMCs, osteoblast/chondrocyte‐like VSMCs, or mesenchymal‐like VSMCs [2]. This process leads to secondary inflammation and creation of even more foam cells, as macrophage‐like VSMCs can take up the lipids and become the cause of the inflammatory response [3].

The inflammation process and oxidative stress in atherosclerosis can also be related to the cellular senescence of endothelium. The cell, whose cell cycle is permanently arrested, is characterized by senescence‐associated secretory phenotype and the release of IL‐6, IL‐1, IL‐8, TNF‐α—inflammatory cytokines. Moreover, the reduced NO and increased ROS levels are observed in the senescent endothelium, which leads to vascular disfunction [24, 25, 26]. The inflammation and thickening of tunica intima leads to the O_2_ local deficiency, also known as hypoxia. In response to hypoxic conditions, the expression of hypoxia‐inducible factor‐1 subunit alpha (HIF‐1α) is increased, which in turn induces the release of vascular endothelial growth factors (VEGFs). VEGFs induce the process called neovascularization, which is the formation of new blood vessels from vasa vasorum, with the aim of nutrients supplying. However, in contrast to the angiogenesis in normal conditions, neovascularization promotes the progression of atherosclerotic lesions [4, 27].

Despite so many new findings, atherosclerosis still remains a deadly condition and requires much research that will reveal potential therapeutic targets. In recent years, a potential role of miRNA in atherosclerosis was discovered. It was reported that miRNA can give both positive and negative effects, as it can arrest the inflammation process, but also is involved in the PI3K/Akt pathway and therefore it inhibits the apoptosis of endothelium cells. Some research also focus around siRNA, especially N‐acetyl galactosamine‐conjugated siRNA, which can bind with the molecules like lipoprotein (a) and thereby reduce the development of the atherosclerotic plaque [4, 28]. There is also a growing interest in the pro‐ and antiatherosclerotic role of anti‐inflammatory, antioxidative, angiogenic cytokine—TGF‐β [28, 29]. In the next section, we are going to describe the most important signal pathways involved in the progression of atherosclerosis.

## 3 Signal Pathways in Atherosclerosis

**FIGURE 3:**
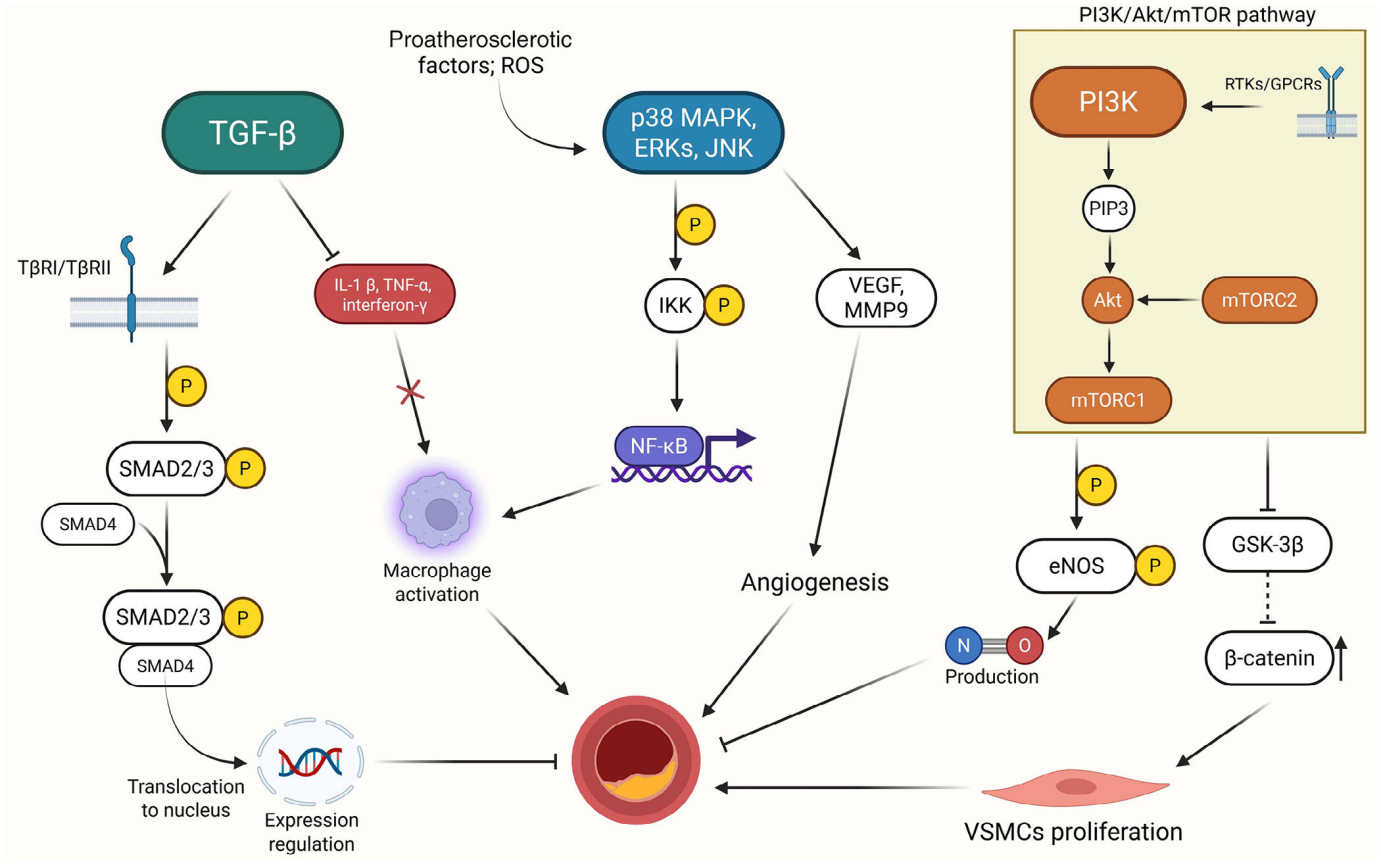
The figure below presents the main signal pathways involved in the atherosclerosis progression. Transforming growth factor beta (TGF‐β) signaling is shown through activation of transforming growth factor beta receptor Type I and Type II (TβRI/TβRII), leading to phosphorylation of SMAD family member 2 and 3 (SMAD2/3), association with SMAD family member 4 (SMAD4), nuclear translocation, and regulation of gene expression, preventing the atherosclerosis progression. Proinflammatory mediators, including interleukin‐1 beta (IL‐1β), tumor necrosis factor alpha (TNF‐α), and interferon gamma (interferon‐γ), are depicted in relation to macrophage activation. Reactive oxygen species (ROS) and other proatherosclerotic factors are shown to activate mitogen‐activated protein kinase signaling, including p38 mitogen‐activated protein kinase (p38 MAPK), extracellular signal‐regulated kinases (ERKs), and c‐Jun N‐terminal kinases (JNKs), leading to activation of inhibitor of nuclear factor kappa‐B kinase (IKK) and nuclear factor kappa‐B (NF‐κB)‐dependent transcription. Vascular endothelial growth factor (VEGF) and matrix metalloproteinase‐9 (MMP9) are presented in association with angiogenesis. The phosphoinositide 3‐kinase pathway (PI3K/Akt/mTOR pathway) is shown as activated by receptor tyrosine kinases and G protein‐coupled receptors (RTKs/GPCRs), leading to phosphatidylinositol‐3,4,5‐trisphosphate (PIP3) generation, activation of protein kinase B (Akt), and signaling through mechanistic target of rapamycin Complex 1 and 2 (mTORC1 and mTORC2). Downstream effects include phosphorylation of endothelial nitric oxide synthase (eNOS) and nitric oxide (NO) production, which protects the endothelial cells. Inhibition of glycogen synthase kinase‐3 beta (GSK‐3β) and stabilization of beta‐catenin (β‐catenin) lead to proliferation of vascular smooth muscle cells (VSMCs) and plaque development (illustration created by BioRender.com).

The inflammatory response in macrophages relies on many interconnected signal pathways composed of a huge number of proteins, cytokines, and other molecules impacting each other in a big inflammatory signaling network [30, 31]. Indirectly, those signal pathways regulate various processes involved in the atherogenesis, primarily secretion of proinflammatory cytokines, foam cell formation, VSMCs phenotypic switching, and endothelial senescence [10]. In this section, we are going to present the most important and best‐discovered signal pathways involved in the process of atherosclerosis progression, emphasizing the most current discoveries that can be useful in the future therapies. All the most important factors are presented in Figure 3.

### 3.1 TGF‐β

Transforming growth factor‐β (TGF‐β) is a cytokine that performs various functions in almost every type of cells. Its significant role in cell proliferation and migration, as well as in homeostasis and immune response, makes it an undeniable factor involved in the atherosclerotic plaque formation [32]. TGF‐β1, the best‐discovered isoform in the context of CVD, works mostly anti‐inflammatorily, as it inhibits IL‐1 β, TNF‐α, and interferon‐γ, therefore reducing the macrophage activation [29, 33, 34]. TGF‐β2 was frequently described to play a protective function in the normal artery wall, whereas the lack of that isoforms correlates with the higher risk of the cardiovascular events. The increased level of collagen and VSMCs count that determine the plaque stability are also correlated with the high level of TGF‐β2 [28, 33, 35]. Moreover, TGF‐β2 can reduce the inflammation, while combined with TGF‐β3 [33]. By binding with the TβRI and TβRII receptors on the membranes of endothelial cells and VSMCs, TGF‐β phosphorylates SMAD2 and SMAD3 proteins. After binding with SMAD4, a SMAD protein complex is translocated to the nucleus, where it can regulate gene expression, therefore protecting the VSMCs from phenotypic switching or macrophages from transforming into the foam cells [28].

However, TGF‐β may also work in a negative way, which is frequently a cause of disruption in its proper functioning. Various mutations, especially in TGF‐β or SMAD2/3 encoding genes, can lead to the VSMCs phenotypic switching to the mesenchymal state [6]. What is more, various isoforms of this factor can be activated by inflammatory cytokines or ROS, and thus increase the oxidative stress and inflammation [28]. Chen et al. observed that the inhibition of endothelial TGF‐β in mice reduces the vascular inflammation and arrests the disease progression, in contrast to the same factor in other cell types that is known for playing an antiatherosclerotic role [36]. Another study by Zhu et al. revealed that miR‐375‐3p, a well‐known tumor suppressor miRNA, promotes endothelial senescence in ApoE−/− mice through the TGF‐β/SMAD pathway, therefore promoting the atherosclerosis progression [37]. Hence, the dual role of TGF‐β in atherosclerosis depends on many factors and still needs to be developed. Both atheroprotective and atherosclerotic functions are extremely important in the disease progression and understanding them can bring a significant step forward in preventing atherosclerotic lesions.

### 3.2 MAPK

A multifunctional, MAPK signaling pathway is involved in cellular processes such like proliferation, differentiation, or cell cycle regulation [38, 39]. All of the three major protein subfamilies: p38, extracellular signal‐regulated kinases (ERKs), and c‐Jun N‐terminal kinases (JNKs), are highly connected with CVD progression, especially with heart failure. Activated by various proatherosclerotic factors, MAPK pathway can promote ox‐LDL uptake by macrophages and their transformation into foam cells, inflammation, or VSMCs proliferation and migration [39]. In the macrophages, MAPK mediates the activation of nuclear factor kappa‐light‐chain‐enhancer of activated B cells (NF‐κB) via the phosphorylation of IkappaB kinase (IκK) [10]. Activated NF‐κB stimulates the secretion of proinflammatory cytokines, like IL‐1α, IL‐1β, or IL‐6, and therefore the intensity of inflammatory response [10]. Moreover, MAPK activated by ROS promotes the upregulation of VEGF and matrix metalloproteinase‐9, the major components responsible for the angiogenesis within the atherosclerotic plaque [40]. In response to ox‐LDL, ERK1/2 proteins mediate the VSMCs proliferation, migration, and phenotypic switching [41, 42]. Hypertrophy of VSMCs can also be promoted via the activation of p38 MAPK, induced by Angiotensin II in the oxidative stress conditions, which can be mediated by TGFβ and PPARγ [43]. Recent findings also indicate that β‐sitosterol reduces proatherosclerotic inflammation, oxidative stress, and lipid accumulation via MAPK signaling [44]. Similarly, asiaticoside, a triterpenoid with antiatherosclerotic potential, was found to suppress the inflammation via MAPK/NF‐κB inhibition [45]. Overall, MAPK signaling, synergistically with other pathways, plays a crucial role in plaque formation.

### 3.3 PI3K/Akt/mTOR

The plaque formation is also strictly connected with the PI3K/Akt/mTOR signal pathway, which plays both protective and harmful role in the progression of atherosclerotic lesions [46]. The activation of PI3K protein by activated receptor tyrosine kinases (RTKs) or G protein‐coupled receptors (GPCRs) creates phosphatidylinositol (3,4,5)‐trisphosphate (PIP3), which mediates in activating Akt. One of the complexes, which create mTOR serine/threonine kinase, mTORC2, is a direct activator of Akt, whereas mTORC1 is activated by Akt and is highly involved in the cell proliferation, apoptosis, and differentiation [47].

The activation of PI3K/Akt/mTOR pathway in the endothelial cells promotes the nitric oxide synthase (eNOS) phosphorylation and its production of NO, which is a key mechanism of homeostasis maintain and the integrity of endothelial cells [46, 47]. However, the mTOR signaling also has its impact on VSMCs proliferation and migration, as it prevents VSMCs apoptosis by inhibiting GSK‐3β, thereby upregulating β‐catenin [46]. Moreover, the Akt activity modulation mediates in the macrophage polarization and modulates the inflammatory response and the ox‐LDL uptake [48].

According to Zheng et al. the PI3K/Akt/NF‐κB pathway is a main target of Yaoshi Tongyuan Tablet, a food‐medicine homology formulation with an antiatherosclerotic potential [49]. The suppression of Akt activity and therefore of NF‐κB phosphorylation reduced the secretion of proinflammatory cytokines and ameliorated dyslipidemia [49]. In recent years, it is very frequently observed that the downregulation of PI3K/Akt, thereby upregulating GSK3β, normalizes lipid profiles and prevents the progression of atherosclerosis [50, 51]. Moreover, the PI3K/Akt/NF‐κB cascade drives the excessive immune activation, whereas the inhibition of its activity leads to the polarization of anti‐inflammatory macrophage phenotype and to the plaque stabilization [52]. The dual role of PI3K/Akt/mTOR signal cascade and its connection with other pathways, such as TGF‐β, constitutes a crucial point of research in recent years and an important target in the topic of atherosclerosis progression.

## 4 The Role of CDKs in Atherosclerosis

CDKs are serine/threonine protein kinases, whose most crucial role is controlling the cell cycle—a string of biochemical and biophysical reactions that leads to replication and division of one cell into two daughter cells. In mammals, the cell cycle consists of four main stages, which are gap phase‐1 (G1), DNA synthesis (S), gap phase‐2 (G2), mitosis (M), and the rest phase G0 [53, 54]. Transitions between each phases are controlled by the mechanisms called checkpoints and require involvement of specific CDKs (CDK4, CDK6, CDK2, CDK1, CDK3) and their binding proteins called cyclins (D, E, A, B, and C). The conjunction of CDK with specific cyclin enables the cell to enter the next phase of the cycle. The G1 stage and G1/S transition are controlled by CDK4/6–cyclin D and CDK2–cyclin E complexes, whereas CDK2 combined with cyclin A controls the progression of the S phase. The S/G2 and G2/M transition points are regulated respectively by CDK1–cyclin A and CDK1–cyclin B complexes [53]. The activation of CDK–cyclin complexes also requires the phosphorylation of CDKs’ threonine residues on the segment called T‐loop, which is often conducted by CDK activating kinase (CAK) [17, 53, 54]. In case of any abnormalities, the cell cycle can be arrested by CDK inhibitors from INK4 family (p16^INK4a^, p15^INK4b^, p18^INK4c^, and p19^INK4d^) and the CIP/KIP family (having three members of p21^CIP1^, p27^KIP1^, and p57^KIP2^) [53, 54].

However, not every CDK protein is responsible for the direct control of the cell cycle. There are also many transcriptional CDKs (CDK7, CDK8, CDK9, CDK10, CDK11, CDK12, CDK13, and CDK19), whose most important role is the regulation of the synthesis of ribonucleic acids (RNA) from deoxyribonucleic acids (DNA), also known as the transcription process [17, 54]. In eukaryotic cells, this process consists of three stages: initiation, elongation, and termination, and is carried out by three classes of RNA polymerases. RNA Polymerase I (RNAP‐I) catalyzes the ribosomal RNA transcription, whereas RNA Polymerase III (RNAP‐III) is responsible for transfer RNAs and partly for small nuclear RNAs (snRNAs) synthesis. The most discovered eukaryotic polymerase is RNAP‐II, which carries out the transcription of messenger RNA (mRNA), as well as most snRNAs and miRNAs [17].

An extremely important role in activation of RNAP‐II is played by CDK7, which enables the transcription initiation, CDK9, which is mediately responsible for starting transcription elongation and mediator kinases: CDK8 and CDK19 [53]. A more detailed description of the mechanisms of action of these kinases is presented in subsections below. We are also going to discuss the importance of CDK5 protein, which is often described as quite untypical CDK, but may constitute a significant target in the treatment of many diseases, including atherosclerosis [17]. A comparison of main biological characteristics and the role in atherosclerosis of untypical and transcriptional CDKs, which we are going to describe below, are summarized in the Table 1.

**TABLE 1:** Comparative profile of atypical and transcriptional CDKs implicated in atherosclerosis.

CDK	Class	Activating partner/complex	Principal molecular role	Atherosclerosis‐linked processes	Inflammatory profile	Evidence in VSMCs	Representative inhibitors	Translational relevance	References
CDK5	Atypical CDK	p35/p25	Phosphorylation of noncyclin substrates involved in cell stress, metabolism and inflammatory signaling	Endothelial senescence, increased oxLDL uptake, macrophage foam‐cell formation, plaque‐promoting inflammation	Predominantly proinflammatory (IL‐10 suppression, NF‐kB activation)	No direct VSMC‐centered evidence summarized	Roscovitine, (R)‐DRF053	High	[16, 55, 56, 57, 58, 59, 60]
CDK7	Transcriptional CDK	Cyclin H–MAT1 (CAK complex)	Transcription initiation and activation of cell‐cycle CDKs	Angiogenesis‐associated endothelial responses; possible link to plaque neovascularization	Indirect/context‐dependent	Not established	THZ1	Low to moderate	[61, 62]
CDK8/19	Transcriptional CDK	Cyclin C–MED12/13 (mediator kinase module)	Context‐dependent regulation of transcription and inflammatory gene programs	Putative effects on lipid metabolism and inflammatory signaling	Proinflammatory tendency through IL‐10 repression	Not established	Senexin A/B, RVU120	Potential	[63, 64]
CDK9	Transcriptional CDK	Cyclin T1/T2 or cyclin K (P‐TEFb)	Transcription elongation of stimulus‐responsive genes	Inflammation, monocyte–endothelium interaction, VSMC proliferation, migration and phenotypic switching	Predominantly proinflammatory (NF‐kB‐dependent programs)	Strong evidence for proliferation, migration and phenotypic switching	Flavopiridol, LDC000067	High	[13, 65, 66, 67]

*Abbreviations*: CAK, CDK‐activating kinase; CDK, cyclin‐dependent kinase; IL‐10, interleukin 10; NF‐kB, nuclear factor kappa B; oxLDL, oxidized low‐density lipoprotein; P‐TEFb, positive transcription elongation factor b; VEGF, vascular endothelial growth factor; VSMCs, vascular smooth muscle cells.

### 4.1 The Role of Cell Cycle Proteins Regulation in Atherosclerosis

For about 30 years, a lot of research has been conducted on the involvement of cell cycle‐regulating proteins in atherosclerosis progression. The role of CDK1/2/4/6, which are classic, cell cycle‐related CDKs, was discovered long before the function of CDK5/9 and other untypical ones. It is proved that cell cycle proteins regulate the proliferation of VSMCs. Most of the research have focused on the activity of CDK2 and its natural inhibitors from CIP/KIP family: p27^KIP1^ and p21^CIP1^ in the proliferation and migration of VSMCs [11].

In normal arteries, as well as during the reparation process, p27^KIP1^ is upregulated. Its downregulation is corelating with high level of CDK2–cyclin E and CDK2–cyclin A expression in an injured arterial wall [12]. Although the upregulation of p21^CIP1^ has not always been observed in normal arteries, its significant role in proliferation of VSMCs is unconvertible. CDK2–cyclin E high level was also detected in the atherosclerotic tissue, which confirms the involvement of cell cycle deregulation in vascular diseases [12, 68]. However, not only CIP/KIP inhibitors are responsible for regulating VSMCs proliferation. A study by Gizard et al. confirmed that a member if INK4 family, p16^INK4a^, arrests the cell cycle in G1/S checkpoint, after being activated by PPARα—a lipid metabolism and inflammation regulator. The upregulation of PPARα stimulated the inhibition of CDK4 and reduced the proliferation of VSMCs [69].

It was also reported that p27^KIP1^ and p57^KIP2^ are targets in increasing neointima formation and VSMCs proliferation by miRNA [70]. This observation was confirmed by Lee et al., who discovered that CDK inhibitors from both CIP/KIP and INK4 families are involved in the regulation mechanism of VSMCs proliferation in hypoxic conditions. The expression of p16^INK4a^, p15^INK4b^, and p21^CIP1^ was downregulated after the activation of miRNA, induced by hypoxia, which promoted the VSMCs proliferation. Despite the hypoxic conditions, the suppression of miRNA led to the upregulation of CDK inhibitors and reduced the proliferation [71]. Another study reported that CDK6, a classic regulator of the G1 phase in the cell cycle, plays a key role in the progression of atherosclerosis, as it is involved in the mechanism of proliferation and apoptosis of VSMCs, regulated by circHIPK3—a circular, noncoding RNA [72]. Several studies have mentioned that CDK1 also has its function in VSMCs proliferation [73].

A recent analysis by Hu et al. brought a fresh look on the role of CDK4 and CDK6 in the pathogenesis of atherosclerosis. It turned out that those kinases do not only affect VSMCs proliferation, but also on the vascular cellular senescence. In ox‐LDL‐induced endothelium, the level of CDK4/6 was downregulated, while p16^INK4a^ encoding gene expression was significantly enhanced. The suppression of vascular aging in mice, fed with high‐fat diet (HFD), correlated with the knockdown of p16^INK4a^. Analogically, palbociclib, a selective CDK4/6 inhibitor, did not treat the atherosclerosis, but accelerated its progression, by enhancing the endothelium senescence. This findings open up new strategies for the treatment of vascular diseases [74].

Although many studies have raised a subject of the involvement of CDK2, CDK4, CDK6, and its inhibitors in atherosclerosis, very few research has focused on the transcriptional and untypical CDKs: CDK5/7/8/19/9. In the subsections below, we are going to describe their role in atherosclerosis pathogenesis, due to the foregoing studies. All the preclinical studies relevant to this topic are presented in the Table 3.

### 4.2 CDK5

The CDK5 is an incredibly unique member of the serine–threonine CDK family. Although it has a very homologous amino‐acid sequence to other CDKs, especially CDK1 (58% homology) and CDK2 (62% homology), it is not a typical CDK protein due to several key differences [55, 75]. The main difference is that CDK5 is not activated by binding with any cyclin, but with other proteins called p35 and p39 [55, 76, 77]. T‐loop phosphorylation, which is obligatory in an activation of the most CDKs, is not required in CDK5 activation [55, 75, 76]. Its catalytic activity also cannot be inhibited by specific CDK inhibitors including p27^KIP1^, p57^KIP2^, and p21^CIP155^. In order to inhibit CDK5 in pathological conditions, the most common therapeutic options are the broad‐spectrum pan‐CDK inhibitors like olomoucine, roscovitine, flavopiridol, AT7519, or dinaciclib [55, 75, 76, 78].

The research conducted for over 30 years has revealed that CDK5, after binding with p39 or p35, plays a key role in the nervous system, especially in neuronal migration, neurite outgrowth, and synaptogenesis [55, 75, 76, 77]. A cleavage of p35 into p25 protein causes hyperactivation of CDK5 and may be behind many disorders of nervous system and serious diseases including Alzheimer's or Parkinson's disease. The expression of p25–CDK5 complex may also cause apoptosis of neurons, as well as disruption of their cytoskeletons and morphological degeneration [79, 80].

However, recent studies showed us that it constitutes a crucial factor in many other processes, for example in angiogenesis [55, 75, 81, 82]. Proangiogenic factors, such as VEGF or fibroblast growth factor, are able to activate CDK5, which leads to remodeling of the endothelial cells’ actin cytoskeleton via GTPase Rac1. This process eventually leads to the migration of endothelial cells and angiogenesis [81]. CDK5 was also found to promote angiogenesis by the regulation of VEGF levels or by phosphorylating a catalytic subunit of γ‐secretase—presenilin, leading to cleavage of Notch1 to NICD. Due to several comprehensive reviews, CDK5 phosphorylates HIF‐1α at serine 687, and thus promotes an angiogenesis process [55, 75, 76]. Those findings eventually led us to its role in pathological conditions, for example, in tumor angiogenesis or in aortic dissection, which is a consequence of apoptotic death of aortic endothelial cells that can be induced by CDK5 [81, 82].

Another important role of CDK5 is its involvement in the lipid/glucose metabolism and the pathogenesis of obesity and Type 2 diabetes. Phosphorylation of peroxisome proliferator‐activated receptor γ (PPARγ) at serine 273 by CDK5 causes changes in the expression of genes responsible for the insulin resistance. This reaction is activated by obesity and it leads to many disorders such as insulin resistance, dyslipidemia, or Type 2 diabetes [55, 83, 84]. Later in this review, we are going to highlight the fact that it also plays a significant role in atherosclerosis, as the suppression of ERKs, mediated by CDK5, and its impact on PPARγ indicates an involvement of CDK5 in mitogen‐activated protein kinase (MAPK) pathway, which plays a crucial role in the inflammatory processes (Figure 1) [43, 84].

CDK5 is also involved in regulation of various aspects of the immune response, primarily in promoting T‐cell survival and motility [55].

#### 4.2.1 The Role of CDK5 in Atherosclerosis

The most untypical CDK, which we are going to discuss, is CDK5. Although it does not belong to the group of classis cell cycle proteins, it plays a very significant role in atherosclerosis progression. A study by Chrousos et al. described a role of glucocorticoids in homeostasis and stress regulation [85]. The researchers also discussed a significant impact of glucocorticoids on maintenance of a proper cardiovascular tone and their involvement in atherosclerosis pathogenesis. Considering the fact that CDK5 regulates the transcriptional activity of these hormones, which contributes to the development of neurodegenerative disorders, suggests that the disfunction of glucocorticoids activity may lead to cardiovascular disorders, including atherosclerosis. However, this correlation is not uniquely determined in the study, which means that more research is needed to confirm that the impact of CDK5 on atherosclerosis is correlated with glucocorticoids activity.

**FIGURE 4:**
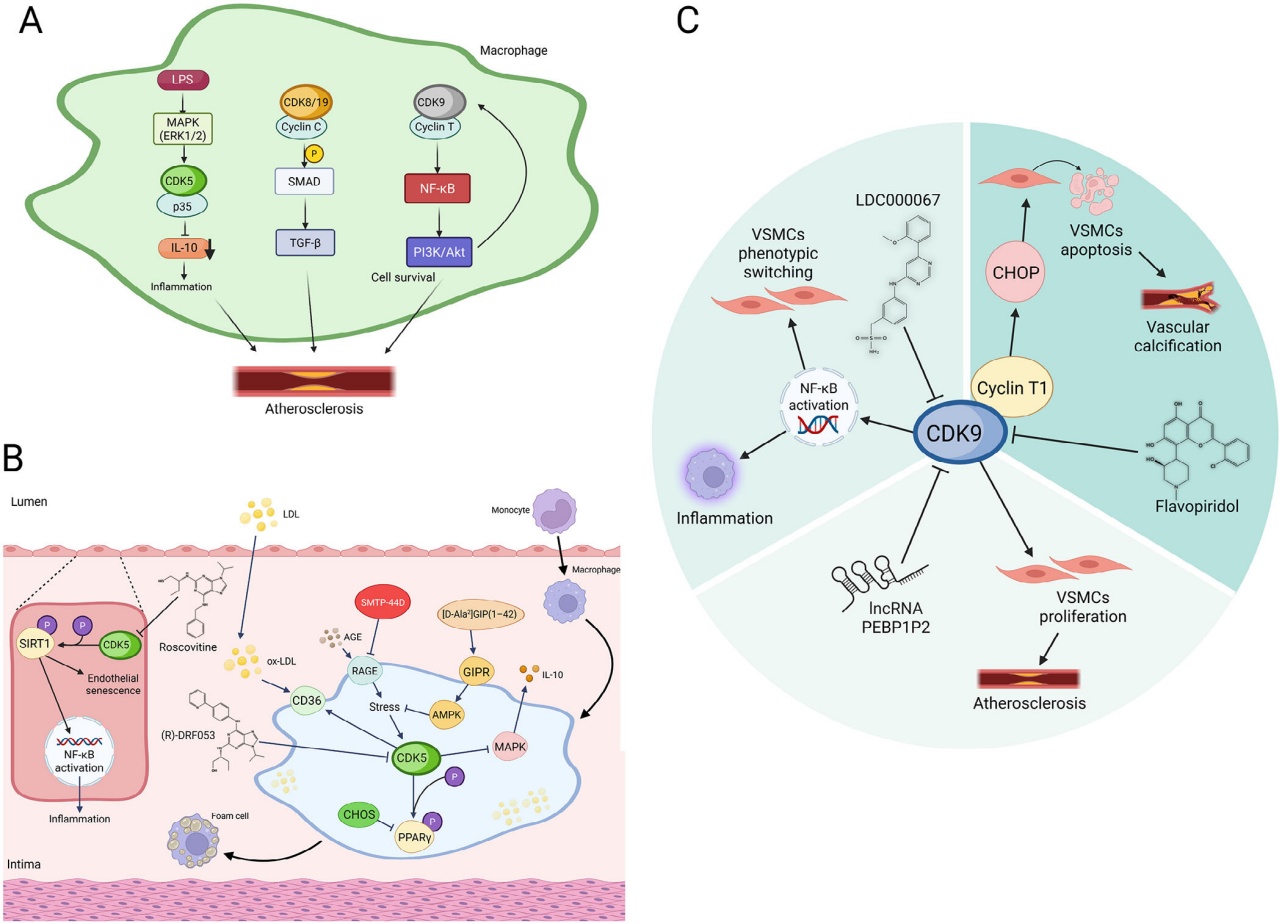
Cyclin‐dependent kinases (CDKs) are observed to be the key regulators in the molecular pathways of atherosclerosis development. (A) CDK5/p35 is activated in macrophages by mitogen‐activated protein kinase (MAPK) pathway and it subsequently suppresses an antiproliferative cytokine interleukin‐10 (IL‐10), promoting a proinflammatory response. CDK8/19 associated with cyclin C phosphorylates SMAD proteins, enhancing transforming growth factor‐β (TGF‐β) signaling. CDK9/cyclin T activates nuclear factor kappa‐light‐chain‐enhancer of activated B cells (NF‐κB), which further supports macrophage cell survival. (B) The phosphorylation of Sirtuin 1 (SIRT1) by CDK5 activates NF‐κB and causes endothelial senescence and inflammation that is inhibited by roscovitine. The oxidized low‐density lipoprotein (ox‐LDL) uptake, mediated by CDK5–CD36 pathway can be arrested by AMP‐activated protein kinase (AMPK), or selective inhibitor (R)‐DRF053. The effect of CDK5–CD36 is related to the oxidative stress generated by advanced glycation end products–receptor for advanced glycation end products (AGE–RAGE), inhibited by SMTP‐44D. CDK5 also phosphorylates peroxisome proliferator‐activated receptor gamma (PPARγ), which is inhibited by CHOS (chitosan oligosaccharide). Another significant role of CDK5 is suppressing MAPK proteins and therefore arresting the IL‐10 secretion. (C) CDK9/cyclin T1 activates C/EBP homologous protein (CHOP), which then promotes the apoptosis of vascular smooth muscle cells (VSMCs) and the vascular calcification, inhibited by flavopiridol. The long noncoding RNA (lncRNA) PEBP1P2 suppresses the CDK9‐mediated VSMCs proliferation. CDK9 is involved in NF‐κB activation, which causes the inflammation and phenotypic switching of VSMCs. The CDK9 activation may be suppressed by novel selective inhibitor LDC000067 (illustration created with BioRender.com).

To our knowledge, a direct correlation between CDK5 and atherosclerosis was first mentioned in a study by Bai et al., who were trying to investigate the molecular mechanism behind endothelial senescence, which frequently leads to atherosclerosis development [15]. They analyzed the cell cultures to find that phosphorylation of sirtuin‐1 (SIRT1), an important vascular aging regulator, is modulated by CDK5 in serine 47 (S47). In vivo, senescent endothelial cells, as well as aortic tissues, were marked with an increased level of CDK5. Inhibition of that kinase decreased SITR1 S47 and arrested endothelial senescence. A significant decrease in atherosclerosis development was observed in mice long‐termed treated with roscovitine, a CDK5 inhibitor. Activation of CDK5 was observed to arrest SIRT1, which normally counters vascular aging and inflammation via NF‐κB pathway (Tables 1, 2, 3 and Figure 4) [15, 56]. The research showed that CDK5 was stimulating the hyperphosphorylation and nuclear accumulation of SIRT1 protein within endothelial cells and eventually led to their senescence. Due to these facts, we can infer that CDK5 can pose a potential target in treating atherosclerosis [56].

**TABLE 2:** Cell type‐specific roles of CDKs in atherosclerosis‐relevant vascular and immune compartments.

CDK	Endothelial cells	VSMCs	Macrophages	Overall implication in atherosclerosis	References
CDK5	Promotes endothelial senescence and inflammatory activation	No direct role established in the cited literature	Enhances foam‐cell formation and suppresses IL‐10	Proatherogenic across endothelial and macrophage compartments	[56, 57, 60]
CDK7	Supports angiogenic endothelial responses	Not established	No direct role established in the cited literature	Possible role in plaque neovascularization rather than core inflammatory remodeling	[62]
CDK8/19	Possible contribution to inflammatory signaling	Not established	Suppresses IL‐10 in myeloid cells	Potential proinflammatory mediator; vascular relevance remains incompletely defined	[64, 86, 87]
CDK9	Promotes inflammatory endothelial–monocyte interaction	Drives proliferation, migration and phenotypic switching	Promotes inflammatory cytokine production	Consistently proatherogenic with the strongest vascular‐wall evidence among transcriptional CDKs	[13]

*Abbreviations*: IL‐10, interleukin 10; VSMCs, vascular smooth muscle cells.

**TABLE 3:** Preclinical evidence for CDK5, CDK7, CDK8/19, and CDK9 in atherosclerosis and related vascular mechanisms.

Study	CDK	Model	Biological system	Principal finding relevant to atherosclerosis	Intervention/inhibitor	References
Bai et al.	CDK5	In vitro and in vivo	Endothelial cells; ApoE−/− mice	CDK5‐mediated SIRT1 hyperphosphorylation promoted endothelial senescence and accelerated atherosclerosis.	Roscovitine	[56]
Na et al.	CDK5	In vitro	LPS‐stimulated macrophages	Early p35–CDK5 activation suppressed IL‐10 and favored a proinflammatory macrophage phenotype.	None	[60]
Yang et al.	CDK5	In vitro (molecular simulation and cell assays)	PPARγ‐related assays	Supported CDK5‐dependent regulation of PPARγ phosphorylation, a pathway with metabolic relevance to atherosclerosis	None	[88]
Choi et al.	CDK5	In vitro and in vivo	Adipose and liver tissues	Linked CDK5‐dependent PPARγ phosphorylation with metabolic dysfunction relevant to atherosclerosis risk	None	[83]
Yashima et al.	CDK5	In vitro	U937 macrophages	AGE–RAGE signaling increased CDK5–CD36 activity, oxLDL uptake, and foam‐cell formation.	(R)‐DRF053	[16]
Terasaki et al.	CDK5	In vitro	U937 macrophages	GIP‐related modulation of CDK5–CD36 signaling reduced foam‐cell formation.	GIP‐related intervention	[58]
Terasaki et al.	CDK5	In vivo and in vitro	ApoE−/− mice; peritoneal macrophages	SMTP‐44D lowered CDK5/CD36/RAGE signaling, reduced AGE‐driven oxLDL uptake, and decreased plaque burden.	SMTP‐44D	[57]
Terasaki et al.	CDK5	In vitro	U937 macrophages	AMPK activation reduced CDK5/CD36 signaling and oxidative stress; protection was reversed by AMPK inhibition.	AMPK‐related intervention	[59]
Gao et al.	CDK7	In vivo	Diabetic vasculopathy mouse model	SHENQI compound improved vascular injury partly through CDK7‐related regulation.	SHENQI compound	[89]
Shi et al.	CDK7	In vitro	HUVECs	VEGF‐induced CDK7 activity supported angiogenesis; CDK7 inhibition suppressed this response.	THZ1	[62]
Song et al.	CDK7	In silico	Human transcriptomic datasets	Identified CDK7 among candidate genes associated with vascular disease progression	None	[90]
Yang et al.	CDK8	In vitro	SREBP‐related assays	Implicated CDK8 in regulation of lipid biosynthesis pathways relevant to atherogenesis	None	[64]
Johannessen et al.	CDK8/19	In vitro	Myeloid cells	Small‐molecule studies identified CDK8 as a negative regulator of IL‐10, supporting a proinflammatory role.	CDK8‐directed small molecules	[91]
Varlamova et al.	CDK8/19	In vivo	Endothelium‐specific CDK8/19 knockout mice	Generated a vascular model supporting future mechanistic work; elevated CDK8 was also noted in injured VSMCs.	Genetic model	[92]
Maturavongsadit et al.	CDK8/19	In vitro and in vivo	Vascular occlusion‐related models	Proposed Senexin A‐based CDK8/19 targeting as a vascular intervention concept	Senexin A	[93]
Han et al.	CDK9	In vitro	Monocytes	CDK9 inhibition reduced cytokine secretion and monocyte–endothelium interaction.	Flavopiridol	[94]
Shiozaki et al.	CDK9	In vitro	VSMCs	CDK9–cyclin T1 promoted CHOP/ATF4 signaling, vascular calcification, and apoptosis.	Flavopiridol	[67]
He et al.	CDK9	In vitro	VSMCs	lncRNA PEBP1P2 suppressed CDK9, reducing VSMC proliferation, migration, and phenotypic switching.	None	[66]
Huang et al.	CDK9	In vitro and in vivo	VSMCs; HFD ApoE−/− mice	CDK9 inhibition suppressed inflammation and VSMC phenotypic switching and attenuated atherosclerosis.	LDC000067	[13]

*Abbreviations*: AGE, advanced glycation end products; AMPK, AMP‐activated protein kinase; ApoE−/− mice, apolipoprotein E‐deficient mice; ATF4, activating transcription factor 4; CHOP, C/EBP homologous protein; GIP, glucose‐dependent insulinotropic polypeptide; HFD, high‐fat diet; HUVECs, human umbilical vein endothelial cells; lncRNA, long noncoding RNA; RAGE, receptor for AGEs; VSMCs, vascular smooth muscle cells.

The function of CDK5 in conjunction with p35 protein is very well known in the nervous system. However, a study by Na et al. proves that CDK5–p35 complex is also involved in inflammatory function in macrophages [95]. CDK5 was found to inhibit the anti‐inflammatory cytokine interleukin‐10 (IL‐10) in macrophages, by decreasing the activation of MAPKs (Tables 1, 2, 3 and Figure 4). The lipopolysaccharide (LPS)‐stimulated macrophages were observed to have a higher level of p35 synthesis and thereby CDK5 activation.

A molecular simulation demonstrated by Yang et al. showed that 5‐cholesten‐3β, 25‐diol, 3‐sulfate (CHOS) may promote inhibition of PPARγ phosphorylation (Tables 1, 3 and Figure 4) [88]. The study also confirmed that the PPARγ phosphorylation at Ser273 is mediated by CDK5 and that PPARγ is a common target in atherosclerosis treatment. PPARγ partial agonists were observed to interact with the β‐sheet region within the PPARγ ligand binding domain and thereby arrest the PPARγ phosphorylation by CDK5. The fact that CDK5 is an important factor in PPARγ phosphorylation was mentioned in older research by Choi et al., where in vitro and in vivo analysis showed a significant correlation between CDK5–PPARγ and obesity/diabetes pathogenesis (Table 3) [83]. Now we are conscious of the fact that it also influences atherosclerosis and other CVDs [88]. CDK5 was also found to incur atherosclerosis by phosphorylating Ser245 of the β‐sheet region of PPARγ ligand‐binding domain (PPARγ‐LBD) [96]. The research suggests a chiral compound, Danshensu Bingpian Zhi, as a potential medication, which is able to protect PPARγ‐LBD from phosphorylating by CDK5.

It was already mentioned before that CDK5, in complex with p35, is involved in inflammation reaction in macrophages stimulated by LPS [95]. A significant progress has been made, when many researchers started concentrating on the correlation between CDK5 and CD36 gene expression, which is known to be involved in the foam cell formation and the acceleration of atherosclerosis pathogenesis in the diabetic conditions [16, 58]. CD36 gene expression in human macrophages is usually increased by advanced glycation end products (AGEs) and related with the ox‐LDL uptake [16, 58]. Yashima et al. observed that the increased ox‐LDL uptake as well as high CD36 and CDK5 coding gene expressions were inducted by AGE in U937 human macrophages. Moreover, both ox‐LDL uptake and CD36 coding gene expression level were arrested by a selective CDK5 inhibitor, (*R*)‐DRF053, which proved that CDK5 can be a used as a biomarker in a treatment of atherosclerotic CVD under diabetic conditions (Tables 1, 2, 3 and Figure 4) [16].

In another study by Terasaki et al., CDK5 gene expression level was arrested by [D‐Ala^2^]GIP(1–42), an agonist for glucose‐dependent insulinotropic polypeptide (GIP) receptor, in human cultured U937 macrophages [57]. The effect was correlated with a suppression of scavenger receptor CD36 expression. CDK5–CD36 pathway was reported to be arrested by GIP, which consequently led to inhibition of foam cell formation of macrophages (Table 3 and Figure 4). (*R*)‐DRF053 targeted at U937 macrophages, gave the similar results to [D‐Ala^2^]GIP(1–42), although its combination with [D‐Ala^2^]GIP(1–42) did not bring any additive results. The atheroprotective role of GIP and its correlation with CDK5 inhibition may constitute a good path in searching for novel therapies for treating atherosclerosis.

Moreover, the expression level of CDK5, along with CD36 and receptor for AGEs (RAGE), was also significantly inhibited in peritoneal macrophages from apolipoprotein‐E null (*ApoE−/−*) mice, treated with SMTP‐44D (Table 3 and Figure 4) [59]. SMTP‐44D is a small‐molecule metabolite, which was reported not only to decrease the surface area of atherosclerotic lesions, but also to reduce macrophage infiltration and AGEs accumulation. The researchers suggest that CDK5–CD36 pathway may constitute a potential target of SMTP‐44D in ox‐LDL uptake by macrophages, induced by AGEs–RAGE axis, which confirms its role in atherosclerosis.

A decrease in CDK5 and CD36 mRNA was also observed in the inhibition of the AGE‐induced ROS generation of U937 macrophages (Table 3 and Figure 4) [97]. The research showed that dorsomorphin, an inhibitor of AMP‐activated protein kinase (AMPK), targeted at AGE‐exposed U937 cells, decreases the action of [D‐Ala^2^]GIP(1–42). Analogically, an AMPK activator was observed to have an antiatherosclerotic effect, similar to [D‐Ala^2^]GIP(1–42). The researchers emphasized a significant role of reducing the CDK5–CD36 pathway by [D‐Ala^2^]GIP(1–42) in U973 macrophages, which was already mentioned in previous studies. They also showed that inhibiting this pathway is associated with arresting the NADPH oxidase‐derived oxidative stress generation, induced by AGE–RAGE.

The impact of CDK5 on ROS generation is an extremely interesting topic, as this relation can be observed on many levels. First, the proatherogenic phosphorylation and inactivation of SITR1 lead to the enhanced oxidative stress and eventually endothelial senescence, which was proved by the in vivo analysis on ApoE−/− mice on HFD, injected with roscovitine [15]. Second, oxidative stress caused by AGE–RAGE enhances CDK5 activation in macrophages, which mediates the foam cell formation [16]. Third, Terasaki et al. observed that the inhibition of NADPH‐oxidize correlates with the lower CDK5 level in macrophages, which decreases ox‐LDL uptake [97]. All those findings indicate that both CDK5 and ROS generation have a significant impact on each other and are involved in the development of atherosclerotic lesions and should be analyzed deeper in order to find the new therapies for CVD treatment.

### 4.3 CDK7

The CDK7, similarly to CDK5, is not a typical member of CDKs family. Instead of controlling the cell cycle directly, like CDK2 or CDK4, it plays a fundamental role in phosphorylating other CDKs in the T‐loop. Its activity is also essential in the process of synthesis of RNA from DNA; hence, it is classified as one of the transcriptional CDKs [17, 61].

A proper progression through the G1 phase in the cell cycle is only possible after binding CDK4 and CDK6 with cyclin D. However, CDK4/6–cyclin D complex cannot be activated without phosphorylation of the threonine residues (Thr172 in CDK4 and Thr177 in CDK6), catalyzed by CDK‐activating kinase (CAK) complex, which consists of CDK7, cyclin H, and ménage‐a‐trois 1 (MAT1) [17, 61]. Analogically, CAK phosphorylates CDK2 in Thr160 and CDK1 in Thr161, in order to activate the CDK2–cyclin E and CDK1–cyclin B complexes, allowing the cell to go through G1/S and G2/M transitions [17, 61].

CAK constitutes a part of the general transcription factor IIH (TFIIH), in which it phosphorylates the carboxyl terminal domain (CTD) of RNAP‐II at serine residue—Ser5. This reaction is essential to start the transcription initiation [17, 53, 61]. CDK7 is also involved in activation of CDK9–cyclin T complex (that controls elongation transcription process), homeostasis, DNA repair process, as well as in the progression of various diseases, including cancer [53, 61]. Although an inhibition of CDK7 is not very promising in monotherapy; it may constitute a potential target in synergistic therapy [61].

#### 4.3.1 A Potential Role of CDK7

There is a hypothetic role of CDK7 in the pathogenesis of atherosclerosis, although the details of the potential mechanisms remain unclear. Geo et al. explored the impact of traditional Chinese medicine compound SHENQI on vascular lesions [89]. SHENQI strongly inhibited diabetic atherosclerosis‐predominant vasculopathy. The therapeutic mechanism of SHENQI's curative effect includes regulation of CDK7.

A recent study by Shi et al. showed a significant role of CDK7 in the process of angiogenesis. The analysis of human umbilical vein endothelial cells (HUVECs) revealed that the level of expression of CDK7 and, catalyzed by this kinase, phosphorylation of RNAP‐II were increased by VEGF, which is known to play a crucial role in angiogenesis [62(p7)]. A selective CDK7 inhibitor, THZ1, enhanced apoptosis and significantly arrested the formation of capillary tube, as well as proliferation and migration inducted by VEGF (Tables 1, 2, and 3). Although this study does not mention the atherosclerosis, it proves the involvement of CDK7 in the process of blood vessels formation and suggests CDK7 as a potential target in vascular diseases, which means that new research are needed to explore this topic in details.

Song et al. conducted an analysis of the genes that may be involved in the progression of atherosclerotic plaque, by using the bioinformatic methods: weighted gene correlation network analysis and the dynamic network biomarker algorithm [90]. The analysis revealed that one of the 13 most important genes, which play a key role in the atherosclerotic plaque progression, is the CDK7 coding gene (Tables 1 and 3). This discovery may be very important in the light of future research of the atherosclerosis and the role of CDK proteins in its pathogenesis.

Although correlation of CDK7 and atherosclerosis has not been mentioned in any other study so far, it can potentially constitute a target in atherosclerosis treatment, considering its similar mechanisms to CDK9 and an important role in transcription regulation by phosphorylating RNAP‐II [61, 62]. Several studies also report the involvement of CDK7 in cardiomyocyte division and proliferation [61, 98].

### 4.4 CDK8/19

CDK8, is a part of the subcomplex of Mediator complex, which is responsible for stabilizing RNAP‐II preinitiation complexes at promoters and thereby regulating the preinitiation process [63]. CDK19 is a paralog of CDK8 protein, as those two proteins have a significantly high homology of their amino acid sequences and their cyclin binding domains are also very similar [17, 53, 99]. The kinase‐module, which consists of CDK8/19, cyclin C, MED12, and MED13, is responsible for phosphorylating a CTD domain of RNAP‐II, thereby disrupting the connection between DNA promoter and polymerase [17, 63]. It can also work as a positive transcription regulator by modulating the activity of various transcription factors, like TFIIH by the phosphorylation of cyclin H, as well as NF‐κB, STATs, or estrogen receptor [53, 100].

CDK8 is also involved in controlling the cell cycle, by regulating the levels of the important cell cycle‐related proteins like p53, p21^CIP1^, and p27^KIP197^. This fact, as well as the involvement in many signaling pathways, highlights CDK8 and CDK19 as the oncogenic drivers and targets in cancers like hepatocellular carcinoma, ovarian cancer, gastric cancer, breast cancer, pancreatic cancer, melanoma, prostate cancer, or acute myeloid leukemia (AML) [17, 100]. In recent years, the research of potential therapies for these conditions brought many CDK8/19 inhibitors, including RVU120, TSN084, and Senexin B, which were involved in Phase I/II clinical trials [100].

The Mediator complex also plays an essential role in a transcription downstream of TGF‐β, by phosphorylating, and therefore regulating, the SMAD protein family [100]. SMAD proteins are directly involved in the TGF‐β signaling, which is responsible for the crucial processes in atherosclerosis, such like cholesterol regulation, plaque stability, and VSMC function. The SMAD‐dependent TGF‐β pathway is also related to PI3K/Akt pathway, which is also involved in many pathological processes in atherosclerosis [28]. It presents the complexity of all signaling pathways, which play their functions in atherosclerosis progression (Figure 4).

Several studies also indicate that CDK8 and CDK19 are indispensable in the epigenetic modifications. There is a growing interest in the topic of CDK8/19‐mediated transcriptional reprogramming by chromatin and enhancers looping [99], but also on histone modification. According to Tsutsui et al., those two kinases interact with the histone arginine methyltransferase PRMT5 and transcriptionally repress the CCAAT enhancer‐binding protein (C/EBP) β target genes, by controlling the symmetric dimethylation of histone H4 arginine 3 in their promotor regions [101]. Because C/EBPβ is involved in immune response, we should consider the role of epigenetic modifications mediated by CDK8/19 in CVD progression.

#### 4.4.1 A Potential Role of CDK8/19 in Atherosclerosis

There were also several studies that suggest a potential role of CDK8/19 in the pathogenesis of vascular diseases. According to study by Yang et al., CDK8 is involved in the regulation of sterol regulatory element‐binding protein (SREBP) transcription factors that play crucial role in lipid biosynthesis (Table 1). As dysregulation of lipid homeostasis is frequently responsible for various CVDs, CDK8 may constitute a potential target in their treatment [64]. Moreover, the analysis of myeloid cells by Johannessen et al. showed that CDK8, along with CDK19, inhibits IL‐10, an anti‐inflammatory cytokine, which suggests that these kinases may be used as targets in the treatment of inflammatory disorders (Tables 1 and 3) [91]. Varlamova et al. created a mouse strain without expression of CDK8/19 in vascular endothelium, by crossbreeding three distinct mouse strains. As the increased level of CDK8 had been observed in the injured VSMCs in mice and in the human atherosclerotic arteries, the researchers report that the new mouse strain may be useful in analyzing the role of CDK8/19 in the pathogenesis of atherosclerosis (Table 3) [92]. There were also mentions that a selective CDK8/19 inhibitor, Senexin A, may constitute a new candidate for treating occlusive vascular disease, a condition closely related to atherosclerosis (Table 3) [93].

According to the comprehensive review by Schiano et al., MED12 and MED13, which are the components of the CDK8 module in the Mediator complex, play the key role in various CVDs, such like aortic dissention or cyanotic heart disease. Moreover, various modules of the Mediator were described to regulate the VSMC proliferation and migration, as well as the TGF‐β pathway, which is highly connected with the development of the atherosclerosis [87, 102]. The recent studies also show that CDK8/19 along with MED1 are deeply involved in the transcription of inflammatory genes, including NF‐κB [103].

To our knowledge, no other research has been conducted since then. This topic, although it is still very poorly discovered, has a potential to be developed and may turn out to be very important in the atherosclerosis treatment. A much better described kinase in terms of its role in the progression of atherosclerosis is CDK9, which is going to be discussed in the next subchapter.

### 4.5 CDK9

Whereas CDK7 controls the transcription initiation, the CDK9 plays a crucial role in the elongation stage of this process [53, 65]. Activation of this kinase takes place after its binding with one of the three T‐type cyclin members: T1, T2a, T2b, or with cyclin K. However, in contrast to described above CDK5, CDK9 requires phosphorylation of the threonine 186 residue in a C‐terminal loop named the T‐loop [17, 65]. CDK9 is a catalytic subunit of positive transcription elongation factor b (P‐TEFb) and, along with cyclin T or K, it constitutes the core of this complex.

The main and most discovered role of P‐TEFb is the phosphorylation of CTD domain of RNAP‐II, though in a different position than CAK. P‐TEFb inhibits the repressive effect of negative transcription elongation factor and 5,6‐dichloro‐1‐β‐d‐ribofuranosylbenzimidazole sensitivity inducing factor, which prevent transcription elongation. Therefore, CDK9–cyclin T allows the cell to exit the checkpoint and enter a productive transcription elongation [17, 53, 65].

CDK9 occurs in eukaryotic cells in two different isoforms, which are originally identified CDK9_42_, also known as 42 kDa form or PITALRE, and 13 kDa larger CDK9_55_. CDK9_42_ is diffused in the nucleoplasm and it is mostly responsible for controlling the transcription process, whereas CDK9_55_ is located in nucleus and is expressed by a TATA‐box. In opposite to CDK9_42_, it is also able to bind Ku70 protein, which suggest its potential involvement in the repair of DNA [17, 104, 105].

P‐TEFb is involved in progression of many diseases, like leukemia and lymphoma, and is regulated by various signaling pathways. In a normally working cell, it is mostly sequestered in a small RNA complex called 7SK snRNP, where its activity is arrested by hexamethylene bisacetamide‐inducible proteins 1 or 2 (HEXIM1 or 2). Releasing of P‐TEFb from this inhibitory complex can be induced by many impacts, mostly by stress response [17, 65, 106]. Another protein that makes a complex with P‐TEFb is bromodomain‐containing protein 4 (BRD4). In this complex, P‐TEFb is catalytically active, because of the specific feature of BRD4, which is binding of acylated histones and transcriptional mediators [17, 106]. A highly important role of CDK9 is the transcription of antiapoptotic factors of B‐cell lymphoma 2 (Bcl‐2) family. One of them is myeloid cell leukemia‐1, high expression of which is widely observed in AML. Others are Bcl‐2 and X‐linked inhibitor of apoptosis, also related to various types of leukemia. Activation of these genes is highly related to PI3K/Akt, as it was discovered that inhibiting CDK9 and PI3K simultaneously inducts apoptosis and prevents leukemia progression [65, 107, 108]. A relation between CDK9 and PI3K/Akt pathway is extremely interesting, as regulation of PI3K–Akt–mTORC1 activity level appears to play a key role in the macrophage polarization and apoptosis, and it constitutes a cause of macrophage survival and inflammatory signaling (Figure 4) [48, 108].

In recent years, many research of CDK9 inhibition were conducted. CDK inhibitors that had been already used in clinical trials are mostly pan‐CDK inhibitors like flavopiridol, dinaciclib, roscovitine, RGB‐286638, or SNS‐032. However, there are few preclinical studies, which show a potential effect of CDK9 inhibitors with high selectivity, like i‐CDK9 [65, 105, 106, 107(p9)]. Particularly noteworthy is LDC000067—a selective CDK9 inhibitor, analyzed, for example, in atherosclerosis, what we are going to prove later in this review [13, 14, 65, 105]. A recent development in novel techniques brought some new alternative methods for CDK inhibition, including PROTACs. PROTACs are the molecules that consist of the E3 ubiquitin ligase recruiting element and the ligand for the protein of interest (such like CDK9). In opposite to CDK inhibitors, PROTACs are not only able to block the protein kinase from proper functioning, but also to make the 26S proteasome recognize and degrade the kinase [109]. This method brought significantly good results in degrading CDK9, with much higher selectivity and effectivity than classic CDK9 inhibitors, like SNS‐032 [110].

#### 4.5.1 The Role of CDK9 in Atherosclerosis

Findings that determined the role of CDK9 protein in the pathogenesis of atherosclerosis have emerged in recent years. Han et al. highlighted the role of CDK9 in the transcription and apoptosis of monocytes, as well as in the secretion of inflammatory cytokines and the interaction with endothelium cells [111]. The inhibition of the CDK9, along with monocyte transcriptional inhibition, was suggested as a potential way to decrease the pathogenesis of atherosclerosis by reducing the inflammation.

Later, the role of CDK9 in the atherosclerosis inflammation was confirmed by research (Table 3) [94, 112]. Serum, monocytes, and artery plaque samples taken from patients with atherosclerotic CAD, where atherosclerosis is considered a crucial factor, were compared with healthy control subjects. Proteomic analysis showed an increased level of CDK9 protein in samples of CAD patients. Moreover, within artery plaque, a high infiltration of CD14^+^, a monocytes/macrophages surface marker, was observed in correlation with CDK9 increased expression. CDK inhibitors targeted at CDK9, including flavopiridol, successfully arrested proliferation of the monocytic acute leukemia cell line (THP‐1).

Another research by Shiozaki et al. revealed a correlation between CDK9–cyclin T1 complex and vascular calcification—a process that can be relevant to many diseases, including chronic kidney disease or atherosclerosis [67]. CDK9 was observed to regulate vascular calcification through induction of proapoptotic transcription factor CCAAT enhancer—binding protein homologous protein (CHOP), which is responsible for VSMCs’ apoptosis, and the phosphorylation of the activating transcription factor 4 (ATF4) pathway. Research showed that within various kinase inhibitors, only those which inhibited CDK9, like flavopiridol, arrested CHOP expression. Although the work is focused mainly on chronic kidney disease, it confirms the significant role of CDK9 in CHOP expression, which in turn is responsible for VSMCs apoptosis and vascular calcification (Tables 1, 3 and Figure 4). This information can have a crucial role in treating CVDs like atherosclerosis.

He et al. described an association between PEBP1P2—a long noncoding RNA (lncRNA) and the dysfunctional proliferation of VSMCs [66]. Bioinformatical analysis supported by RNA immunoprecipitation assays showed that PEBP1P2 is directly targeted at CDK9. When PEBP1P2 was knocked down, western blotting and immunofluorescence detected an increased level of CDK9 expression, which was later found to regulate the proliferation, migration, and phenotypic transformation of VSMCs (Tables 1, 3 and Figure 4). Cognately, PEBP1P2 upregulation led to CDK9 decreased expression. Inhibiting CDK9 in VSMCs not only slowed down the rates of proliferation and migration, but also upregulated the expression of contractile genes, including α‐smooth muscle actin, calponin 1, and smooth muscle myosin heavy chain.

In another study, Huang et al. analyzed mouse models with atherosclerosis induced by HFD, in order to determine the role of CDK9 in the pathogenesis of this disease [13, 14]. The level of CDK9 protein was notably increased in the serum and in the aortas of the mice fed with HFD. The mice were later treated with a highly specific small‐molecule CDK9 inhibitor, LDC000067. In vivo, CDK9 inhibitor consequentially arrested inflammation, cell proliferation, and phenotypic switching of VSMCs and had an analogous effect on ox‐LDL‐treated human VSMCs in vitro. It is worth noting that LDC000067 had no significant impact on serum lipid profile induced by HFD, which means that CDK9 protein has no involvement in lipid metabolism. In vitro analysis also showed that inhibiting CDK9 by LDC000067, as well as silencing by siRNA, is able to arrest ox‐LDL‐induced inflammation, proliferation, and phenotypic switching of VSMCs. Moreover, further studies demonstrated that CDK9 mediates in the development of atherosclerosis via NF‐κB signaling pathway (Tables 1, 2, 3 and Figure 4). LDC000067 reduced the translocation of NF‐κB subunit p65 to the nuclear from the cytoplasm and therefore inhibited ox‐LDL‐induced NF‐κB activation in VSMC.

### 4.6 CDK‐Targeted Therapy Limitations

However, despite the fact that targeting CDKs is a promising option for atherosclerosis therapy, there are also a few important contraindications, which should be mentioned. Many CDK inhibitors are unselective, including roscovitine, which is commonly used for targeting CDK1/2/5/7 and 9. The low selectivity is often associates with inhibiting not only the kinases, which are involved in the progression of a disease, but also with disrupting the work of many other kinases, essential for the proper cell division and transcription [54]. Another problem related to the clinical use of CDK inhibitors is cardiotoxicity. Even the selective CDK inhibitors, such like palbociclib or ribociclib, targeted at CDK4/6, were reported to incur heart failure and hypertension [113, 114]. This is why one of the biggest challenges in looking for novel potential CDK‐targeted therapies is to produce the CDK inhibitors, standing out with possibly highest selectivity and low toxicity. This problem is still accurate and needs to be included in the future research.

### 4.7 CDKs in Atherosclerosis: Future Directions

To sum up, both cell cycle‐regulating and transcriptional/atypical CDKs seem to play various functions in the progression of atherosclerotic lesions. Although every CDK regulate different molecular pathways, the main modulator appears to be CDK5. According to the present knowledge, it has no significant expression changes in the VSMCs, like it is in the case of CDK9, but it is a master regulator of macrophage transformation into foam cells. Several studies have proved its essential role in the AGE/RAGE pathway and the regulation of ox‐LDL uptake by CD36 receptor. Another kinase competing for the title of the main regulator of atherosclerosis is CDK9, due to its involvement in the VSMCs’ proliferation and migration. CDK9 might even be better option for the target therapy than CDK5, as it has already passed the first preclinical analysis on the human‐derived cells, with low toxicity. However, its direct place in the pathological signal pathways is less clear than in the CDK5 case. The future studies should focus accordingly on analyzing the cytotoxicity and efficiency of CDK5 inhibition, and the molecular mechanism of CDK9, for the better understanding of its direct function.

The broad, complicated molecular landscape standing behind atherosclerosis still forces the researchers to look for more detailed signal pathways and more advanced therapy strategies. In this review, we have mainly focused on the potential of cell cycle proteins, pointing their significant role in various processes linked to atherosclerosis. However, we have also mentioned the limitations of the therapies based on CDK targeting, like cardiotoxicity [113]. As in an anticancer therapy, some level of toxicity is tolerable; due to the essence of the patients’ survival itself, in the atherosclerosis treatment, it could be too dangerous.

However, not all CDKs constitute useful targets only in oncology. In opposite to CDK4/6, a kinase extensively discussed in this work, CDK5, is a common target in the neurodegenerative diseases [55]. Furthermore, CDK9 has been analyzed as a target in cardiology [48, 65, 107]. This fact suggests the CDK5/9 inhibition as more efficient and safer options for an antiatherosclerotic strategy. Another solution to the toxicity of CDK inhibitors may constitute the nanoparticle encapsulation [115, 116]. Nanoparticles may reduce the negative side effects by helping the inhibitors penetrate directly to the plaque in the arteries, without attacking the healthy tissues. Moreover, after the crucial clinical studies, the inhibitors may be replaced by the protein‐degrading PROTACs technology, which will be broadly described in the next section. However, it is possible that these strategies will be permanently replaced by other therapeutic methods, such as immunosuppression or clonal hematopoiesis, as these technologies have been developing rapidly in recent years [117, 118].

## 5 Novel Therapeutic Interventions

**FIGURE 5:**
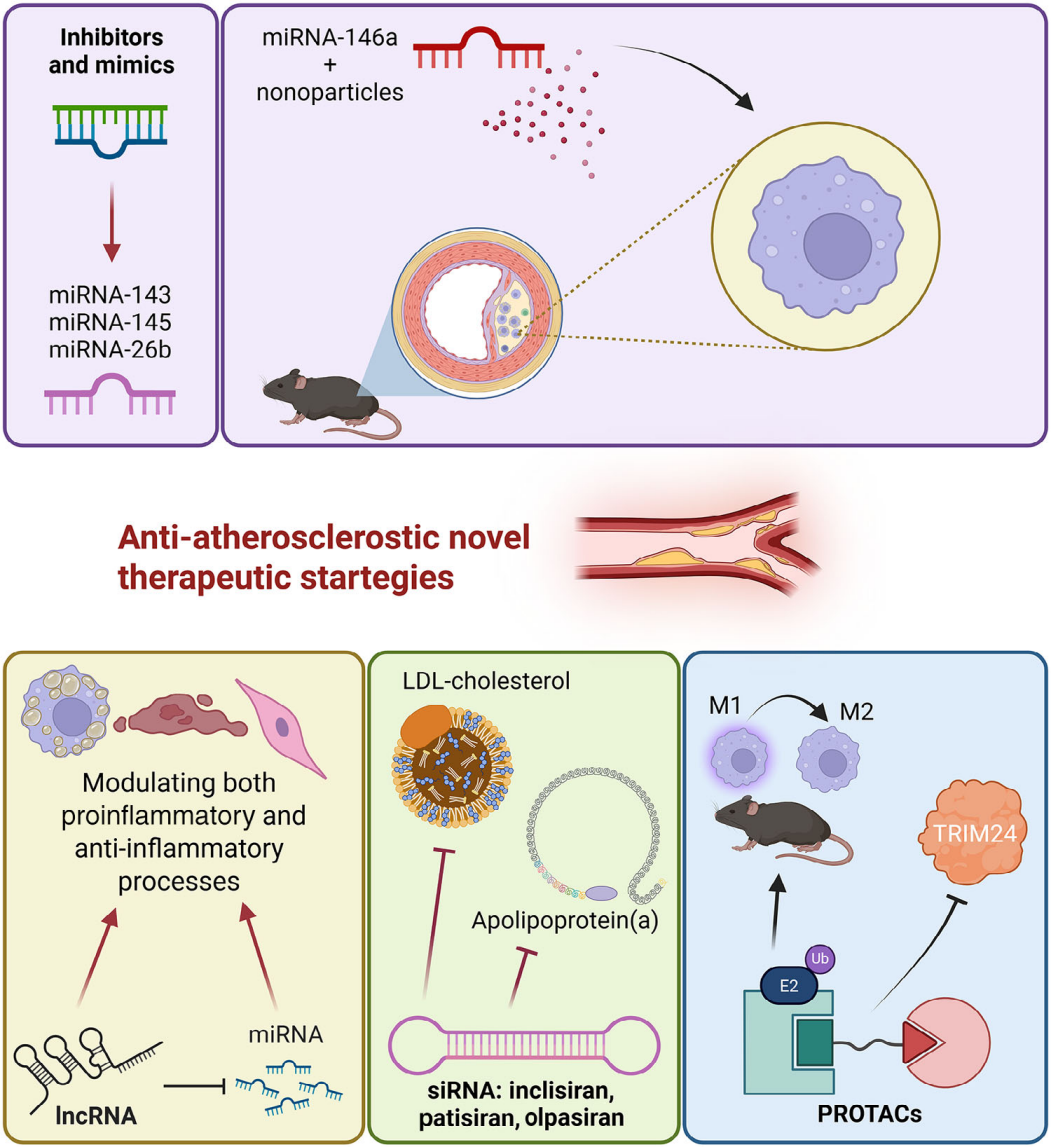
The figure presents novel therapeutic strategies against atherosclerosis. The inhibitors and mimics, targeted at various microRNA (miRNA), can increase or decrease the expression levels of miRNA‐143, miRNA‐145, miRNA‐26b, therefore decreasing the inflammation and atherosclerosis progression. The figure consists of graphical interpretation of the experiment using miRNA‐146a and nanoparticles to lower the inflammation response within the plaque macrophages in mice. Long noncoding RNA (lncRNA) may stimulate the expression of various miRNA, therefore modulating the atherosclerosis phenotype elements, what makes it a good therapeutic strategy. The therapies based on small interfering RNA (siRNA), including drugs like inclisiran, patisiran, or olpasiran, were proved to decreasing the LDL‐cholesterol and apolipoprotein(a) levels. Another novel strategy, proteolysis targeting chimeras (PROTACs) were found to promote the macrophage phenotype switching from proatherosclerotic (M1) to antiatherosclerotic (M2), by targeting and degrading the tripartite motif containing 24 (TRIM24) protein (illustration created with BioRender.com).

The current therapeutic interventions against atherosclerosis mainly focus around lipid lowering by delivering drugs, such like statins, fibrates, or proprotein convertase subtilisin kexin Type 9 (PCSK9) [119]. Statins are first‐line prescribed drugs, known mainly for stabilizing the plaque with thickened fibrous caps, whereas fibrates, like fenofibrates, reduce the level of triglycerides, apoB, or LDL‐C [120, 121]. However, although the LDL‐receptor regulator PCSK9 was proved to impact the key mediators of inflammation, all above drugs work mainly by lipid reducing [122, 123]. In recent years, the need of finding new therapeutic methods, which include targeting the proinflammatory factors or arresting the VSMCs phenotypic switching, is significantly growing [119]. Therefore below, we summarize the potential of novel antiatherosclerotic therapeutic interventions and the importance of future clinical trials on their safety and efficacy. Figure 5 presents the graphical scheme of the most important novel antiatherosclerotic strategies.

### 5.1 RNA‐Based Strategies

Recently, the therapeutic strategies based on targeting specific RNA have emerged as a highly promising option for atherosclerosis treatment. Given their key role in macrophage metabolism, inflammation, and foam cell formation, non‐coding regulatory RNAs are emerging as promising CVD biomarkers and therapeutic strategies [124, 125]. Herein, we briefly describe the significant role of miRNAs, lncRNAs, and siRNAs in future antiatherosclerotic strategies.

#### 5.1.1 MiRNAs

The potential of targeting the noncoding, counting about 20 nucleotides, miRNAs has been deeply discovered in recent years [126]. MiRNAs were found to influence various pathological processes, which are involved in the formation of the atherosclerotic plaque, by modulating target gene expression [127, 128]. The VSMCs proliferation and phenotypic switching was frequently observed to be modulated by miRNA‐143 and miRNA‐145, which made those miRNAs being analyzed as the potential biomarkers of atherosclerosis [126, 129]. Many miRNAs were found to have influence on the endothelial dysfunction, VSMCs proliferation, or foam cell formation via various biological pathways, including NF‐κB, MAPK, or TGF‐β/Smad [127]. According to the recent research, miRNA‐155 increases the oxidative stress by inhibiting eNOS, thereby reducing NO production, eventually leading to endothelium dysfunction [130]. In turn, miRNA‐26b plays an atheroprotective function by stimulating collagen breakdown and reducing the proinflammatory cytokines secretion [131].

In recent years, the nanomaterial‐based electrochemical biosensors were analyzed as the potential method of detecting miRNA biomarkers in blood samples [132]. Various studies have focused on targeting miRNAs with novel drugs in purpose of suppressing the progression of CVD. Many research have pointed out the antiatherosclerotic effects of salvianolic acid, sodium butyrate, or icariside, which protect endothelial cells from apoptosis and reduce lipid accumulation [127, 133]. The best‐discovered method of targeting miRNAs is the application of selective inhibitors and mimics, synthetic RNA‐imitating molecules, in order to reduce or induce the expression of miRNAs [134]. There is also a growing interest in using nanotechnology to deliver the miRNAs, which are known to play the antiatherosclerotic functions. Li et al. have designed the specific nanosystem, containing polydopamine nanoparticles doped with arginine and gadolinium ions, with the function of delivering miRNA‐146a to plaque macrophages [135]. This solution effectively reduced the inflammation and eliminated ROS within the vessel wall, leading to stabilization of the atherosclerotic plaque, proving the great potential of using nanotechnology and miRNAs in atherosclerosis treatment. The list of the miRNA‐based clinical research in atherosclerosis is presented in Table 4.

**TABLE 4:** A list of ongoing and clinical trials relevant to the use of miRNA therapies in CVD treatment.

NCT number	Status	Phase	Objectives	References
NCT05680935	Completed	Not applicable	Effect of miRNA and trimethylamine N‐oxide on atherosclerotic lesions	[136]
NCT03855891	Completed	Not applicable	Determining the spectrum and levels of miRNAs in different stages of atherosclerosis	[137]
NCT04089943	Completed	Not applicable	Exploring the mechanisms which miR‐210 regulates oxidative stress in peripheral artery disease	[138]

#### 5.1.2 LncRNAs

Beside the miRNAs, the lncRNAs, which consist of 200 or more nucleotides, are also involved in CVD risk factors modulation [139]. LncRNAs are responsible for the regulation of various epigenetic modifications, transcription, or splicing events, inside and outside the nucleus. They are much more tissue specific than miRNAs and they can impact other forms of RNA, including mRNA or miRNAs, thereby modulating their availability and expression [134, 139]. Some lncRNAs were reported to promote pathological processes like endothelial dysfunction or VSMCs migration, other, to prevent them and protect the vessel walls [140, 141].

According to the recent research, lncRNAs can influence the NF‐κB signal pathway and modulate inflammation [142]. However, similarly to miRNAs, different lncRNAs can modulate the atherosclerosis progression through different biological pathways. The growth arrest‐specific 5 lncRNA was found to promote the atherosclerotic lesions by interacting with proteins and acting as a competing endogenous RNA [140]. Huang et al. observed that CARMN lncRNA regulates the VSMCs autophagy via Akt/autophagy‐related 7 pathway [143]. In turn, the colon cancer‐associated transcript 1 lncRNA was identified by Zhang et al. to interact with miRNA‐296‐3p, thereby promoting the ox‐LDL uptake by macrophages and inflammatory response [144]. Novel therapeutic methods of targeting lncRNAs using antisense oligonucleotides, like Gapmer ASOs, can modulate the expression or modify the splicing by binding the target RNA [145]. Therefore, ASOs or siRNA, which we are going to discuss in the next paragraph, can modulate the vascular condition by, for example, reducing LDL cholesterol [141]. However, more preclinical research are needed to confirm it.

#### 5.1.3 SiRNA

In recent years, there has been a growing interest in the novel therapeutic strategy called siRNA. This technique is a more innovative alternative to the protein inhibition. It uses modified, small, double‐strand RNA molecules that bind to the mRNA sequences and reduce the protein production [146]. Several novel drugs, including inclisiran, patisiran, or olpasiran, have been used in clinical trials in CVD therapy, as they work in various antiatherosclerotic ways, for example, by reducing LDL cholesterol or apolipoprotein(a) [147]. The siRNA nanoparticles designed by He et al. were able to interfere with IRF5, a molecule in macrophages responsible for plaque destabilization. In ApoE−/− murine models, IRF5 siRNA reduced the proinflammatory genes expression in macrophages and stabilized the plaque in blood vessels [148]. Other studies using siRNA nanoparticle platform were performed by Huang et al. and Tao et al., who targeted the Ca^2+^/calmodulin‐dependent protein kinase γ (CaMKIIγ), in order to arrest the atherosclerosis progression [149, 150]. In the mouse model, CaMKIIγ siRNA was observed to decrease the necrotic area and increase the phagocytosis of apoptotic cells in plaques, reducing the atherosclerosis progression [150]. The most accurate clinical trials, which used siRNA drugs, mainly inclisiran, as a treatment strategy for atherosclerosis, were placed in the Table 5.

**TABLE 5:** A list of ongoing and clinical trials relevant to the use of siRNA therapies in CVD treatment.

NCT number	Status	Phase	Objectives	Preliminary findings	References
NCT05030428	Active, not recruiting	III	The benefits of inclisiran on major adverse cardiovascular events in patients with CVD	—	[151]
NCT05537571	Completed	II	Efficacy, safety, and tolerability of SLN360 in patients with elevated lipoprotein(a) level and high risk of CVD	The drug was well tolerated and reduced lipoprotein(a) concentration.	[152]
NCT03060577	Completed	II	Long‐term dosing of inclisiran and participants with high CV risk and elevated LDL‐cholesterol	The drug was well tolerated and reduced LDL cholesterol.	[153]
NCT06431763	Completed	IV	Comparison of LDL‐cholesterol lowering with inclisiran and bempedoic acid	—	[154]
NCT04765657	Active, not recruiting	III	Efficacy and safety of inclisiran in Asian patients with CVD	Inclisiran was effective and safe.	[155]
NCT04270760	Completed	II	Effect of olpasiran in patients with CVD	Olpasiran reduces lipoprotein(a) level with good efficiency and safety.	[156]
NCT03705234	Active, not recruiting	III	Effects of inclisiran on clinical outcomes in patients with CVD	—	[157]
NCT05888103	Completed	III	Effects of inclisiran on Chinese adults who were not on any lipid lowering therapy	—	[158]
NCT02597127	Completed	II	Effects of inclisiran on LDL‐cholesterol lowering	The drug was effective.	[159]
NCT06586684	Not yet recruiting	IV	Effect of inclisiran on carotid plaques as assessed by carotid ultrasound	—	[160]
NCT03399370	Completed	III	Effects of inclisiran on LDL‐cholesterol lowering	The drug was effective.	[161]
NCT03400800	Completed	III	Effects of inclisiran on LDL‐cholesterol lowering	The drug was well tolerated and safe.	[162]

*Abbreviations*: CVD, cardiovascular diseases; LDL, low‐density lipoprotein.

### 5.2 PROTACs

PROTACs, molecules consisting of an E3 ligase recruiter, a protein binder and a chemical linker, constitute a huge step forward in the targeted protein degradation therapy [109]. This novel technique has been analyzed in the direction of future atherosclerosis treatment via polarization of M1 (proinflammatory) to M2 (anti‐inflammatory) macrophages [163]. PROTAC constructed by Huang et al. was targeted at tripartite motif containing 24 (TRIM24), a protein that arrest M2 macrophage polarization via Stat6 acetylation [164]. Additionally, poly (lactic‐co‐glycolic acid) nanoparticles were used for PROTAC degrader encapsulation. Thanks to this nanomaterial, a degrader showed a significant specificity for M1 macrophages, without attacking those with M2 phenotype. In ApoE−/− mice, macrophages were significantly polarized into M2 phenotype, which led to the arrest of atherosclerosis progression.

Furthermore, PROTACs method was used by Dong et al. to degrade hexokinase 2, an important enzyme in aerobic glycolysis, in order to arrest the lactate increase. The degradation silenced the histone H3 lysine 18 lactylation transcription, thereby proving the significant relation between histone lactylation and atherosclerosis progression via the endothelial‐to‐mesenchymal transition [165]. Moreover, Li et al. demonstrated a significant impact of the tumor necrosis factor receptor‐associated protein 1 (TRAP1), a metabolic ageing regulator, on VSMCs senescence, by degrading TRAP1 with PROTACs [166]. The same method was later used to target methyltransferase‐like protein 4, a specific enzyme of the N6‐methyldeoxyadenosine (6mA) of mammalian mitochondrial DNA (mtDNA), to confirm the role of mtDNA 6mA in atherosclerosis [167].

Therefore, the use of PROTACs in the future atherosclerosis treatment seems to be very promising, especially because of the significant selectivity and low toxicity. The interest in this method grows extremely fast, as it is leads not only to the protein inhibition, but to its complete degradation. The ongoing and future clinical trials should be conducted in order to confirm the safety and efficacy of PROTACs in combination with nanotechnology [168].

## 6 Conclusions

The molecular pathogenesis of atherosclerosis seems to be very complex. The number of known signal pathways involved in the immune response, endothelial senescence and VSMCs proliferation, migration and phenotypic switching, is still getting bigger. TGF‐β, MAPK, and PI3K/Akt/mTOR are probably the best‐discovered biological pathways working in both protective and atherosclerotic way.

The transcriptional and untypical CDKs may constitute the targets of the future atherosclerosis therapies. What we find interesting is the fact that both CDK5 and CDK9 performed the functions in the inflammation process, but among these two kinases, only CDK9 was involved in the proliferation of VSMCs. Although the role of CDK7 in the formation of atherosclerotic plaque still requires confirmation, its involvement in angiogenesis process constitutes a good starting point to future research. Analogically, the function of CDK8/19 in lipid metabolism indicates the probable impact of those two kinases in the progression of atherosclerotic lesions. It seems to be worth noting that different CDKs does not play their functions at the same stadium of the disease progression. CDK5 is probably most active in the early phase, as it constitutes a driving force behind such processes like endothelium senescence or ox‐LDL uptake, while CDK9, which is involved in VSMCs phenotypic switching and migration, is probably more active in the later stadiums of atherosclerosis. However, more research on that topic is needed.

It is important to note that most of the findings mentioned in this review were presented on the in vitro (isolated macrophages, VSMCs, endothelial cells) or in vivo (like ApoE−/− mice) models. There are very few current clinical trials, which would present an entire effect of CDK inhibitors on human organism. Also, the molecular pathways may be slightly different in the cells of mice than in human cells. Moreover, we cannot identify the role of CDKs on the whole atherosclerosis pathophysiology by the analyses on the isolated cells. This fact is worth considering during the planning of the future studies, which should include the role of CDKs in the human organism and the direct effects of CDK inhibitors on the patients.

In alternation to the classic, pharmacological CDK inhibition, knockdown of its coding genes with siRNA or silencing its activity appear to be extremely promising methods. Silencing the CDK9 protein with RNA‐based interventions effectively reduces endothelial inflammation and oxidative stress, therefore arresting the development of atherosclerotic lesions. Future studies should also include degrading various transcriptional and untypical CDKs, which are hard to be selectively inhibited, with the novel PROTACs method that may lead to the less side effects than classic small‐molecule inhibitors. PROTACs is a very promising strategy, as it not only arrests the protein function, but completely degrades it. Moreover, it seems to be a good option for atherosclerosis treatment as it has been already preclinically in the macrophage M1/M2 phenotype polarization. However, the clinical trials are necessary to confirm its safety and efficacy in patients. Another novel therapeutic intervention is noncoding RNA, including miRNA and lncRNA targeting. Silencing them with siRNA combined with nanomaterials significantly reduces the inflammation and foam cell formation.

Despite the unclarity of many processes, especially those associated with CDK7 and CDK8/19, the rare CDKs may constitute promising targets in the treatment of vascular diseases. The preclinical and clinical analysis should be conducted possibly quickly, especially on CDK5 and CDK9 in order to find new therapy methods.

## Author Contributions

All authors contributed to the study conception and design. Conceptualization was performed by Agnieszka Żuryń and Maciej Gagat. The original draft of the manuscript was written by Jonatan Kaszubski, Agata Wawrzyniak, Maciej Gagat, and Agnieszka Żuryń. Figures were done by Jonatan Kaszubski. All authors commented on previous versions of the manuscript. All authors read and approved the final manuscript.

## Funding

The present study was cosupported by research tasks within the framework of statutory activities (Nicolaus Copernicus University in Toruń, Faculty of Medicine, Collegium Medicum in Bydgoszcz, Poland).

## Ethics Statement

The authors have nothing to report.

## Conflicts of Interest

The authors declare no conflicts of interest.

## Data Availability

No new data were created or analyzed in this study. Data sharing is not applicable to this article.
